# Efficacy and Safety of Safinamide as an Adjunct to Levodopa for Motor Fluctuations in Parkinson’s Disease: A Systematic Review and Meta‐Analysis of Randomized Controlled Trials

**DOI:** 10.1155/padi/9352886

**Published:** 2026-10-06

**Authors:** Ali Abdelhaleem Omar Ahmed, Omer Abdalhaleem Omer Ahmed, Afrah S. Dishean, Collins Gilbert, Malaz Mohsin Anbar Sabah Elkheir, Henry A. Sota, Mohamed Adil Mubarak Abdulaziz, Eman Yasir Mohamed Mahmoud, Rana Abdelhaleem Omar Ahmed

**Affiliations:** ^1^ Department of Medicine National Ribat University Faculty of Medicine Khartoum Sudan duhs.edu.pk; ^2^ Department of Medicine University of Khartoum Faculty of Medicine Khartoum Sudan duhs.edu.pk; ^3^ Department of Medicine St George’s University Saint George Grenada duhs.edu.pk; ^4^ Department of Medicine National University – Sudan Khartoum Sudan duhs.edu.pk

**Keywords:** levodopa, meta-analysis, motor fluctuations, Parkinson’s disease, safinamide, systematic review

## Abstract

**Background:**

Motor fluctuations often occur with long‐term levodopa treatment in Parkinson’s disease (PD). Safinamide is a drug that inhibits monoamine oxidase B (MAO‐B) and modulates glutamate. It is used together with levodopa. A summary of the latest evidence is needed to assess its effectiveness and safety.

**Objective:**

To assess the effectiveness and safety of safinamide as an additional therapy to levodopa for motor fluctuations in PD.

**Methods:**

A systematic review and meta‐analysis followed PRISMA guidelines. A thorough literature search was done from the beginning until January 2026 across PubMed, Scopus, the Cochrane Central Register of Controlled Trials, and Google Scholar. We included randomized controlled trials (RCTs) that compared safinamide (100 mg/day) to placebo as an addition to levodopa in adults with PD and motor fluctuations. The primary effectiveness outcomes were the change in daily OFF‐time (in hours) and the Unified Parkinson’s Disease Rating Scale Part III (UPDRS‐III) score. The main safety outcome was the occurrence of dyskinesia. We assessed the risk of bias using the Cochrane RoB 2 tool. Data were combined using random‐effects models, with results shown as mean difference (MD) or risk ratio (RR) with 95% confidence intervals (CIs). We evaluated the certainty of evidence using GRADE. The protocol was registered with PROSPERO (CRD420261298124).

**Results:**

Three RCTs (*N* = 1127 participants) were included. Safinamide 100 mg significantly reduced daily OFF‐time compared to placebo, with an MD of −1.21 h (95% CI: −1.60 to −0.82; *I* ^2^ = 39%; moderate certainty). It also significantly improved motor function, as shown by the UPDRS‐III MD of −3.13 points (95% CI: −4.89 to −1.37; *I* ^2^ = 72%; low certainty). However, safinamide increased the risk of dyskinesia, with a RR of 3.01 (95% CI: 1.92 to 4.70; *I* ^2^ = 0%; moderate certainty). In a secondary analysis of the Hattori trial’s 50 mg arm (*n* = 131), safinamide 50 mg reduced OFF‐time by −1.25 h (95% CI: −1.92 to −0.58) and improved the UPDRS‐III by −4.06 points (95% CI: −5.54 to −2.59). This effect was smaller for OFF‐time compared to the 100‐mg dose, suggesting a potential dose–response relationship that warrants further investigation.

**Conclusion:**

Safinamide 100 mg per day appears to be a helpful addition to levodopa for reducing OFF‐time and improving motor function in PD patients with motor fluctuations. However, this benefit comes with a higher risk of dyskinesia, so careful patient selection and monitoring are important. The 50‐mg dose shows similar but less pronounced effects, supporting 100 mg as the preferred dose. Further research is needed to confirm these findings.

## 1. Introduction

Parkinson’s disease (PD) is a progressive neurodegenerative disorder characterized by motor symptoms such as bradykinesia, rigidity, and tremor, which result from the loss of dopaminergic neurons in the substantia nigra [1, 2]. The disease has a prolonged prodromal phase, during which nonmotor symptoms and subtle biomarker changes precede the classical motor diagnosis [3]. It imposes a significant and growing burden on healthcare systems globally [4]. Levodopa, the most effective pharmacological therapy for motor symptoms, provides significant symptomatic relief. However, its long‐term use is frequently complicated by the development of motor fluctuations, including “wearing‐off” phenomena and debilitating dyskinesias [5, 6]. These complications arise from the progressive loss of dopaminergic terminals, leading to nonphysiological, pulsatile dopamine stimulation [5]. As the disease advances, an estimated 40%–50% of patients will experience these fluctuations within 4–6 years of levodopa initiation [6], representing a major clinical challenge that significantly impairs quality of life.

To manage motor fluctuations, adjuvant therapies are added to levodopa to stabilize its pharmacokinetic profile and extend its therapeutic effect. These include catechol‐O‐methyltransferase (COMT) inhibitors, monoamine oxidase B (MAO‐B) inhibitors, and dopamine agonists [7]. Safinamide, a novel drug with a dual mechanism of action, offers a distinct approach. It functions as a selective, reversible MAO‐B inhibitor, prolonging synaptic dopamine action while modulating abnormal glutamate release via sodium channel blockade [8, 9]. This unique profile suggests robust efficacy for motor function while possibly offering a different side‐effect profile compared to purely dopaminergic agents.

Several previous systematic reviews and meta‐analyses have evaluated safinamide in PD. Kulisevsky et al. conducted a thorough review of safinamide’s effects on both motor and non‐motor symptoms. They concluded that safinamide improves motor fluctuations and may help specific non‐motor areas [10]. Other combined analyses have shown that safinamide is effective when added to levodopa. However, to our knowledge, no previous meta‐analysis has specifically focused on the approved 100 mg/day dose to provide a GRADE‐assessed synthesis of both efficacy and safety. Additionally, we present a secondary descriptive analysis of the 50‐mg dose from the Hattori trial to provide exploratory data on dose–response relationships.

The 100 mg per day dose is the approved treatment dose for managing motor fluctuations in PD, according to both the US Food and Drug Administration (FDA) and the European Medicines Agency (EMA). The 50‐mg dose is sometimes used as a starting dose or in milder cases, but it is considered too low for motor outcomes based on dose–response data from key trials [11]. Therefore, this meta‐analysis focuses on the important 100‐mg dose while also presenting descriptive data from the 50 mg group in the Hattori trial as a secondary analysis [12].

We conducted this systematic review and meta‐analysis to gather the latest evidence from randomized controlled trials on the effectiveness and safety of safinamide 100 mg/day as an additional treatment with levodopa for motor fluctuations in PD. We focused on patient‐centered and clinician‐assessed efficacy outcomes, including daily OFF‐time and the Unified Parkinson’s Disease Rating Scale Part III (UPDRS‐III) score. We also considered the important safety outcome of dyskinesia to offer a balanced and relevant assessment for practitioners.

## 2. Methods

### 2.1. Search Strategy

A systematic literature search was conducted across PubMed, Scopus, Cochrane Central, and Google Scholar from inception to January 2026, in accordance with the PRISMA 2020 guidelines. The protocol for this systematic review was registered with PROSPERO (PROSPERO Registration Number: CRD420261298124).

The search strategy was developed using the Population, Intervention, Comparison, Outcome (PICO) framework. It included combinations of keywords and controlled vocabulary (MeSH, Emtree). The core concepts were “Parkinson’s Disease” [Mesh], “safinamide” [Supplementary Concept], “Levodopa” [Mesh], and “Motor Disorders” [Mesh] combined with terms for clinical trials. The search was limited to studies involving humans. The reference lists of included articles and relevant reviews were screened to identify additional eligible studies. The study selection process is outlined in the PRISMA 2020 flow diagram (Figure 1).

**FIGURE 1:**
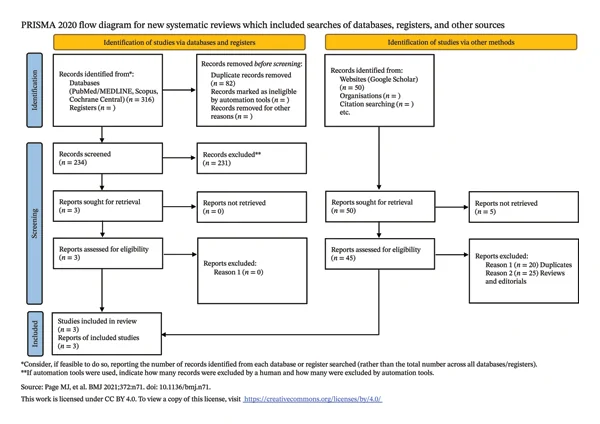
PRISMA flow diagram. Duplicates were removed in two stages: 82 duplicates from 316 database records and 20 duplicates from 50 gray literature/registry records.

### 2.2. Eligibility Criteria

Eligible studies were randomized controlled trials (RCTs) with a parallel‐group or cross‐over design. The population included adult patients (≥ 18 years) diagnosed with idiopathic PD who had motor fluctuations despite stable levodopa therapy. The intervention was oral safinamide at a therapeutic dose of 100 mg/day, used alongside levodopa. This dose was chosen because it is the approved therapeutic dose for managing motor fluctuations according to the US FDA and the EMA. The 50‐mg dose is considered too low for improving motor outcomes based on dose–response data from key trials, so it was not included in the primary analysis. However, descriptive data from the 50 mg arm of the Hattori trial are included as a secondary analysis. The comparator was a placebo with levodopa.

The main effectiveness outcomes were the change from baseline in daily OFF‐time (hours) and the change in the UPDRS‐III motor score (points). The main safety outcome was the occurrence of dyskinesia. While OFF‐time was not the designated primary endpoint in all included trials, it was consistently reported as a key secondary or diary‐derived outcome and was selected as the primary outcome for this meta‐analysis due to its direct clinical relevance and consistent reporting across studies. All three trials utilized standardized 24‐h patient diaries to record ON/OFF states, ensuring comparability of OFF‐time measurement. Studies were limited to those published in English.

Studies were excluded if they were not RCTs, involved nonhuman subjects, used safinamide alone, or did not report on any of the prespecified outcomes of interest.

### 2.3. Data Extraction and Management

Two reviewers independently screened all records and extracted study data using a pre‐piloted form. Disagreements were resolved by consensus or consultation with a third reviewer.

The extracted data included the following:

- Study characteristics: first author, year, design, country, setting, funding, and trial identifier.
- Participant characteristics: sample size, age, sex, disease duration, baseline OFF‐time, and UPDRS‐III scores.
- Intervention details: safinamide dose, titration, concomitant medications, and study duration.
- Outcomes data: For continuous outcomes such as OFF‐time and UPDRS‐III, the mean change from baseline, standard deviation (SD), and number of participants (N) for each group at the primary endpoint were extracted. For dichotomous outcomes, event counts and group totals were documented.

For the Hattori trial, which reported least squares mean differences (MDs) with standard errors, the SDs for the change from baseline were calculated using the formula SD = SE × √N. This conversion was applied specifically to the OFF‐time outcome data.

The characteristics of the included trials are summarized in Table 1. All extracted data were verified against the original publications for accuracy.

**TABLE 1:** Characteristics of included studies.

Study (year), country	Design	Participants (N)	Intervention (dose)	Control	Duration (weeks)	Primary outcome(s) reported
Schapira et al. (2017), Multinational [13]	RCT, double‐blind, placebo‐controlled, parallel group	Saf: 274 Pbo: 275 Total: 549	Safinamide 50 mg/day, increased to 100 mg/day after 14 days	Matching placebo	24	Change in daily ON‐time without troublesome dyskinesia (hours)
Hattori et al. (2020), Japan [12]	RCT, double‐blind, placebo‐controlled, parallel group, phase II/III	Saf 50 mg: 131 Saf 100 mg: 128 Pbo: 136 Total: 395 (FAS)	Safinamide 50 mg/day or 100 mg/day (fixed dose)	Matching placebo	24	Change in daily ON‐time without troublesome dyskinesias (hours)
Wei et al. (2022), China (XINDI study) [14]	RCT, double‐blind, placebo‐controlled, parallel group, phase III	Saf: 151 Pbo: 154 Total: 305 (Safety Set)	Safinamide 50 mg/day for 2 weeks, then 100 mg/day	Matching placebo	16	Change in mean total daily OFF‐time (hours)

Abbreviations: FAS, full analysis set; Pbo, placebo; RCT, randomized controlled trial; Saf, safinamide.

### 2.4. Quality Assessment

Two reviewers assessed the methodological quality of the included RCTs separately. They used the Cochrane RoB 2 tool. This tool evaluates five key areas: randomization, deviations from intended interventions, missing outcome data, outcome measurement, and selective reporting. Disagreements between reviewers were resolved through discussion or consultation with the third reviewer. An overall judgment of “low risk,” “some concerns,” or “high risk” of bias was assigned to each study.

### 2.5. Statistical Analysis

We used RevMan 5.4 for all analyses. Random‐effects models were applied throughout. For binary outcomes, we pooled risk ratios (RRs) using the Mantel–Haenszel method; for continuous outcomes, we used inverse‐variance weighting to calculate MDs, both with 95% confidence intervals (CIs).

Heterogeneity across trials was assessed using Cochran’s Q (*χ* ^2^) test and quantified by the *I* ^2^ statistic. *I* ^2^ values of 25%, 50%, and 75% were interpreted as indicating low, moderate, and substantial heterogeneity, respectively. A prespecified sensitivity analysis for all primary outcomes was performed using a leave‐one‐out approach to evaluate the influence of individual studies on the pooled estimates and heterogeneity.

### 2.6. Certainty of Evidence Assessment

We applied the GRADE approach to rate the certainty of evidence for each outcome. Two reviewers independently performed the GRADE assessments, and any discrepancies were resolved through discussion to reach consensus. Judgments for each domain (risk of bias, inconsistency, indirectness, imprecision, publication bias) were synthesized to produce an overall certainty rating (high, moderate, low, or very low). The summary of Findings is in Table 2.

**TABLE 2:** GRADE summary of findings.

Outcome; no. of participants (studies)	Anticipated absolute effects (95% CI)	Relative effect (95% CI)	Certainty of evidence (GRADE)	Comments
Daily OFF‐time (hours); *N* = 1127 (3 RCTs)	Safinamide reduced OFF‐time by 1.21 h more than placebo (0.82–1.60 more)	MD ‐1.21 (−1.60 to −0.82)	⊕⊕⊕◯ MODERATE	Downgraded for risk of bias
UPDRS Part III score (points); *N* = 1127 (3 RCTs)	Safinamide improved UPDRS‐III by 3.13 points more than placebo (1.37–4.89 more)	MD ‐3.13 (−4.89 to −1.37)	⊕⊕◯◯ LOW	Risk of bias. Substantial heterogeneity (*I* ^2^ = 72%)
Risk of dyskinesia; *N* = 1127 (3 RCTs)	86 more cases per 1000 (from 42 to 146 more)	RR 3.01 (1.92–4.70)	⊕⊕⊕◯ MODERATE	Downgraded one level for imprecision

## 3. Results

### 3.1. Study Selection

A systematic search of electronic databases, including PubMed, Scopus, and the Cochrane Central Register of Controlled Trials, found 316 records. After removing 82 duplicates, we screened 234 records based on their titles and abstracts. We excluded 231 records, leaving 3 full‐text articles to assess for eligibility. A separate search on Google Scholar found 50 records. Of these, 5 could not be retrieved. The remaining 45 records were assessed for eligibility, of which 20 were duplicates and 25 were reviews or editorials, yielding no additional studies. All 3 full‐text articles from the database searches met the inclusion criteria. The study selection process is summarized in the PRISMA 2020 flow diagram (Figure 1).

### 3.2. Study Characteristics

Three randomized controlled trials (RCTs) met the inclusion criteria for this systematic review and meta‐analysis, comprising a total of 1127 patients (safinamide: *n* = 557; Placebo: *n* = 570) with PD experiencing motor fluctuations on stable levodopa therapy. The included studies were the international SETTLE study [13] (safinamide as add‐on to levodopa in PD with motor fluctuations), the Japanese study [12], and the Chinese XINDI study [14] (XINDI: a phase III trial of safinamide in Chinese PD patients). Treatment duration was 24 weeks for two trials [12, 13] and 16 weeks for the XINDI trial [14]. All studies administered safinamide at a target dose of 100 mg/day as an adjunct to levodopa. The overall risk of bias was judged as having “some concerns” for all three studies, primarily due to potential deviations from intended interventions related to permitted dose adjustments. The characteristics of the included trials are summarized in Table 1.

### 3.3. Efficacy Outcomes

#### 3.3.1. Daily OFF‐Time

The meta‐analysis of the change from baseline in daily OFF‐time demonstrated a statistically significant reduction in favor of safinamide compared to placebo. The pooled MDwas −1.21 h (95% CI: −1.60 to −0.82; *p* < 0.00001), indicating that safinamide reduced OFF‐time by over an hour more than placebo. Statistical heterogeneity was low (*I* ^2^ = 39%; *p* = 0.19) (Figure 2). All three individual studies showed consistent, statistically significant benefits (Hattori 2020: MD −1.72 h; Schapira 2017: MD −1.02 h; Wei 2022: MD −1.10 h).

**FIGURE 2:**
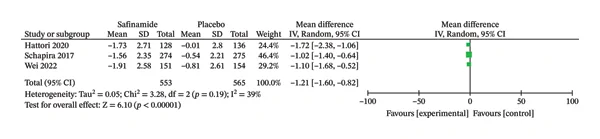
Forest plot: effect of safinamide versus placebo on change in daily OFF‐time (hours).

#### 3.3.2. Motor Function (UPDRS Part III)

Adjunctive safinamide therapy was associated with a significantly greater improvement in motor examination scores. The pooled analysis showed a mean reduction in the UPDRS Part III score that was 3.13 points greater with safinamide than with placebo (95% CI: −4.89 to −1.37; *p* = 0.0005). Considerable statistical heterogeneity was observed across the studies (*I* ^2^ = 72%) (Figure 3). The treatment effect was most pronounced in the Hattori 2020 study (MD −4.18 points), followed by Wei 2022 (MD −3.80 points) and Schapira 2017 (MD −1.60 points).

**FIGURE 3:**
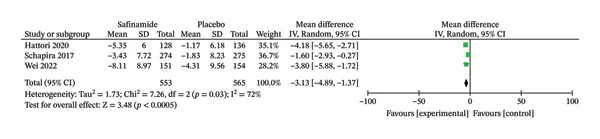
Forest plot: Effect of safinamide versus placebo on change in UPDRS part III motor score (points).

### 3.4. Safety Outcome

#### 3.4.1. Incidence of Dyskinesia

The risk of developing dyskinesia as an adverse event was significantly higher in patients treated with safinamide. The pooled RR was 3.01 (95% CI: 1.92 to 4.70; *p* < 0.00001), indicating a threefold increase in risk compared to placebo (Figure 4). The results were highly consistent across all three trials, with no statistical heterogeneity (*I* ^2^ = 0%; *p* = 0.66). The absolute risk increase, derived from the study control event rates, was approximately 8.6%. It is important to note that this outcome reflects dyskinesia reported as an adverse event at any severity. While dyskinesia was more frequently reported in safinamide‐treated patients, the proportion of patients withdrawing from studies due to adverse events was similar between groups in the included trials [12–14].

**FIGURE 4:**
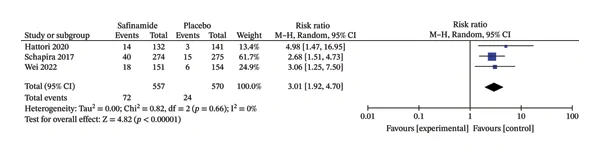
Forest plot: effect of safinamide versus placebo on risk of dyskinesia.

### 3.5. Secondary Analysis–Hattori 2020 50 mg Arm

The Hattori trial included a separate 50 mg safinamide arm (*n* = 131) which was not pooled with other studies due to the absence of a comparable 50 mg arm in the remaining trials. In this single‐study analysis, safinamide 50 mg reduced daily OFF‐time by −1.25 h (95% CI: −1.92 to −0.58) compared to placebo and improved UPDRS Part III score by −4.06 points (95% CI: −5.54 to −2.59). The risk of dyskinesia was increased (RR 3.88; 95% CI: 1.11–13.6). These exploratory findings were directionally consistent with the 100‐mg dose findings, though the comparison is limited to a single trial and should be interpreted as hypothesis‐generating.

### 3.6. Risk‐of‐Bias Assessment

The methodological quality of the three included studies was assessed using the Cochrane Risk of Bias 2 (RoB 2) tool, evaluating five domains: randomization process (D1), deviations from intended interventions (D2), missing outcome data (D3), measurement of the outcome (D4), and selection of the reported result (D5). Overall, the risk of bias was judged as “Some concerns” across all studies. Specifically, Schapira et al. (2017) and Hattori et al. (2020) were rated as “Some concerns” in domains D2 and D5, and D2 and D3, respectively. Wei et al. (2022) was rated as “Some concerns” only in domain D2, with all other domains judged as “Low.” No study was rated as “High” risk of bias in any domain (Figure 5). The consistent concerns mainly arose from potential biases related to deviations from intended interventions, reflecting limitations in blinding or protocol adherence.

**FIGURE 5:**
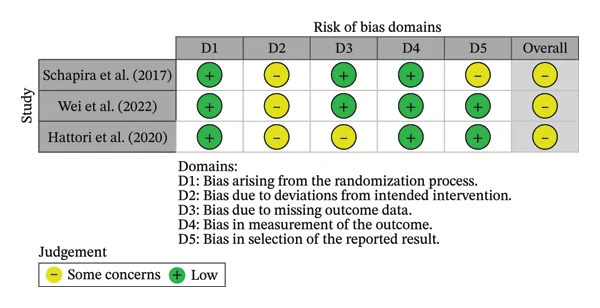
Risk of bias assessment of included randomized controlled trials using the Cochrane RoB 2 Tool.

### 3.7. Sensitivity Analyses

To test the stability of our results, we conducted leave‐one‐out sensitivity analyses. For the reduction in daily OFF‐time, which demonstrated moderate heterogeneity (*I* ^2^ = 39%) in the main analysis, the exclusion of the study by Hattori et al. (2020) [12] yielded a pooled estimate of −1.05 h (95% CI: −1.38 to −0.72; *p* < 0.00001) with zero heterogeneity (*I* ^2^ = 0%). The pooled estimate remained stable, ranging from −1.05 to −1.21 h across all models (Figure 6).

**FIGURE 6:**
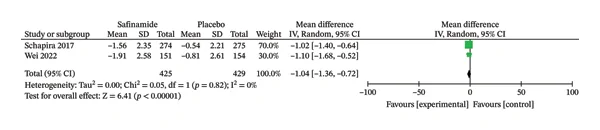
Sensitivity analysis: effect of safinamide on daily OFF‐time after excluding Hattori et al. [12].

For the improvement in UPDRS Part III score, where substantial heterogeneity was observed (*I* ^2^ = 72%), the exclusion of the international study by Schapira et al. [13] was particularly informative. The analysis of the remaining two Asian studies [12, 14] produced a larger pooled MD of −4.05 points (95% CI: −5.25 to −2.85; *p* < 0.00001) with no residual heterogeneity (*I* ^2^ = 0%). This suggests that the observed heterogeneity in the main analysis may be attributable to differences between study populations (Asian vs. international), though the direction and statistical significance of the treatment benefit remained consistent across all sensitivity models (Figure 7). The sensitivity analysis for the incidence of dyskinesia was not performed as the primary analysis already demonstrated perfect homogeneity (*I* ^2^ = 0%).

**FIGURE 7:**
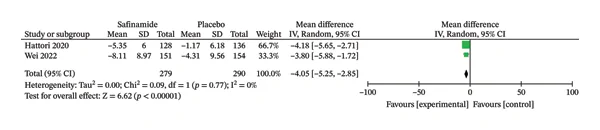
Sensitivity analysis: effect of safinamide on UPDRS Part III score after excluding Schapira et al. [13].

### 3.8. Assessment of Certainty of Evidence

The certainty of the evidence for each outcome was evaluated using the GRADE approach and is presented in Table 2. The evidence for the reduction in daily OFF‐time was graded as Moderate, downgraded one level due to risk of bias (“some concerns” across all studies). The evidence for improvement in UPDRS Part III score was graded as Low, downgraded once for risk of bias and once for substantial inconsistency (high heterogeneity, *I* ^2^ = 72%). The evidence for the increased risk of dyskinesia was graded as Moderate, downgraded one level for imprecision due to wide CIs that cross below the large‐effect threshold of RR 2.0, despite the point estimate of 3.01.

## 4. Discussion

This systematic review and meta‐analysis of three high‐quality RCTs, involving 1127 patients, provides strong and updated evidence that safinamide is an effective and generally safe additional treatment for reducing motor fluctuations in PD. Our findings confirm that safinamide significantly decreases daily OFF‐time by about 1.2 h and improves motor function by more than three points on the UPDRS Part III scale compared to placebo. However, this benefit comes with a threefold higher risk of dyskinesia.

The observed reduction in OFF‐time (MD −1.21 h) is clinically significant. This means patients experience more than an hour of extra functional ON‐time each day. Such a change can greatly affect daily activities and quality of life. This effect size is generally comparable to that of other established adjunctive therapies. For example, rasagiline, another MAO‐B inhibitor, showed a mean reduction of 0.94 h in the LARGO trial [15]. Opicapone, a COMT inhibitor, has also shown similar reductions in meta‐analyses [16, 17]. However, these are indirect comparisons from different studies with varying patient populations, baseline characteristics, and designs. They should not be interpreted as evidence of superiority of any single agent. The motor improvement (MD −3.13 points on UPDRS‐III) is likewise consistent with effects seen with other add‐on agents like istradefylline [7, 18], reinforcing the role of safinamide in treating fluctuating PD. The consistency in effect size supports safinamide’s role as a viable option in the treatment paradigm.

A key finding of our analysis is the significant increase in the risk of dyskinesia (RR 3.01). It is crucial to clarify that this risk refers to dyskinesia reported as an adverse event, meaning its occurrence at any severity during the trial. This is an expected effect of increasing dopaminergic tone and aligns with the mechanism of other dopaminergic adjuncts [7]. However, this finding should not be misinterpreted as a worsening of clinically significant or troublesome dyskinesia. In the included trials, there was no significant increase in severe dyskinesia, and the proportion of patients withdrawing due to adverse events was similar between the safinamide and placebo groups [12–14]. This is clinically important, as the unique glutamatergic modulation of safinamide may theoretically offer a more favorable dyskinesia profile compared to pure dopaminergic agents, an effect that may be better captured by specific severity scales rather than simple adverse event reporting. The risk highlights the importance of selecting patients carefully and dosing them accurately, especially those with a history of or susceptibility to dyskinesia, but it is usually manageable. The long‐term extension study by Borgohain et al. showed that outcomes related to dyskinesia could stabilize, and safinamide’s effectiveness was maintained over 2 years [19], a result confirmed by later real‐world studies [20]. This distinction between any dyskinesia (AE reporting) and clinically significant dyskinesia is a key contribution of our GRADE‐assessed synthesis, providing clinicians with a more nuanced understanding of safinamide’s safety profile.

When considering safinamide among treatment options, its unique dual mechanism is important. Unlike pure dopaminergic agents, safinamide’s additional glutamate modulation may play a role in its effectiveness and could affect non‐motor symptoms. However, our analysis mainly focused on essential motor outcomes [8, 21]. The international MDS evidence‐based review identifies safinamide as useful for managing motor fluctuations [7], and our updated quantitative analysis supports this finding. The choice between additional therapies, such as COMT inhibitors like opicapone [22], other MAO‐B inhibitors, or safinamide, often depends on individual patient factors, side effects, cost, and clinician experience. This principle of selecting treatment based on the trade‐offs between effectiveness and safety has also been highlighted in other medical fields, like lymphoma. Clinicians need to consider response rates compared to toxicity profiles when deciding between immunotherapies [23]. The methodological approaches used in recent systematic reviews across different clinical fields, including emergency medicine, have similarly employed rigorous PRISMA‐guided frameworks to synthesize evidence [24].

Sensitivity analyses were carried out to check the strength of our findings. For daily OFF‐time, which showed moderate variation (*I* ^2^ = 39%) in the main analysis, the leave‐one‐out analysis showed stable results. Excluding any single study resulted in pooled estimates between −1.05 and −1.21 h, all statistically significant, confirming a steady treatment effect. For UPDRS‐III, where variation was high (*I* ^2^ = 72%), excluding Schapira et al. lowered the variation to *I* ^2^ = 0% and yielded a larger pooled estimate of −4.05 points (95% CI: −5.25 to −2.85) from the two Asian trials that remained [12–14]. This result suggests that differences related to the population or study design could affect the variation. However, the small number of trials (*n* = 3) limits formal subgroup analysis, and this interpretation should be seen as exploratory. Recent meta‐analyses in surgical populations have similarly faced challenges with small evidence bases when conducting sensitivity analyses [25]. Similar challenges with small evidence bases and indirect comparisons have been noted in other therapeutic areas, including cardiovascular medicine, where indirect treatment comparisons are frequently used to compare drug classes [26]. The direction and significance of the benefit stayed consistent across all sensitivity models.

As a secondary analysis, the Hattori 2020 trial included a separate 50 mg group. Safinamide 50 mg reduced OFF‐time by −1.25 h (95% CI: −1.92 to −0.58) and improved UPDRS‐III by −4.06 points (95% CI: −5.54 to −2.59). The effect on OFF‐time reduction was similar in direction but smaller in magnitude compared to the 100‐mg dose (−1.72 h). These findings align with the expected dose–response relationship for MAO‐B inhibition and support using the 100‐mg dose as the best therapeutic option for motor fluctuations.

Our review has several strengths. These include following PRISMA 2020 guidelines, using the Cochrane RoB 2 tool for a thorough risk‐of‐bias assessment, and applying the GRADE framework to evaluate the certainty of evidence [27–29]. All included trials were multicenter, double‐blind studies of high quality. The certainty of evidence for OFF‐time reduction was rated as Moderate, downgraded due to risk of bias. For dyskinesia, the rating was moderate, and for UPDRS‐III improvement, it was Low, downgraded due to risk of bias and inconsistency.

Key limitations must be recognized. The number of RCTs included was small (*n* = 3), which prevented a formal assessment of publication bias. We observed substantial differences (*I* ^2^ = 72%) in the UPDRS‐III analysis, likely due to variations in study populations, baseline disease severity, and accompanying medications. Although the overall estimate remained significant, this indicates that the treatment effect on motor function may differ among patient subgroups. Lastly, the follow‐up duration of the included trials (16–24 weeks) only captures medium‐term outcomes. Long‐term efficacy and safety depend on extension studies [19] and observational data [20].

## 5. Conclusion

This meta‐analysis suggests that safinamide is an effective addition to levodopa. It significantly reduces OFF‐time and improves motor function in PD patients with motor fluctuations. However, this benefit comes with a higher risk of dyskinesia, which is reported as an adverse event. Still, this does not seem to lead to a significant rise in severe dyskinesia or in study dropout rates. Exploratory results from the 50‐mg dose group suggest a potential dose–response relationship, though this finding is based on a single trial and requires confirmation. These findings support safinamide as a valuable treatment choice, but more direct comparisons and long‐term data are necessary.

**Nomenclature**
AE: Adverse event
CI: Confidence interval
COMT: Catechol‐O‐methyltransferase
GRADE: Grading of Recommendations, Assessment, Development, and Evaluations
MAO‐B: Monoamine oxidase B
MD: Mean difference
PD: Parkinson’s disease
PRISMA: Preferred Reporting Items for Systematic Reviews and Meta‐Analyses
RCT: Randomized controlled trial
RoB 2: Revised Cochrane Risk‐of‐Bias Tool for Randomized Trials
RR: Risk ratio
SAE: Serious adverse event
SD: Standard deviation
UPDRS: Unified Parkinson’s Disease Rating Scale
UPDRS‐III: Unified Parkinson’s Disease Rating Scale Part III (Motor Examination)

## Author Contributions

Ali Abdelhaleem Omar Ahmed contributed to conceptualization, methodology, project Administration, writing–original draft, writing–review and editing, and formal analysis. Omer Abdalhaleem Omer Ahmed contributed to data curation, formal analysis, and validation. Rana Abdelhaleem Omar Ahmed contributed to data curation, investigation, and visualization. Afrah S. Dishean participated in the investigation and validation. Henry A. Sota and Collins Gilbert extracted and summarized data and critically reviewed the manuscript for important intellectual content. Malaz Mohsin Anbar Sabah Elkheir and Mohamed Adil Mubarak Abdulaziz contributed to literature screening, data extraction, and manuscript review. Eman Yasir Mohamed Mahmoud assisted in data interpretation and preparation of the final manuscript.

## Funding

This research received no specific grant from any funding agency in the public, commercial, or not‐for‐profit sectors.

## Disclosure

All authors read and approved the final version of the manuscript.

## Ethics Statement

The authors have nothing to report.

## Consent

The authors have nothing to report.

## Conflicts of Interest

The authors declare no conflicts of interest.

## Supporting Information

Additional supporting information can be found online in the Supporting Information section.

## Supporting information


**Supporting Information** Supporting File 1 (Data_extraction_sheet.xlsx): Complete data extraction sheet containing raw data from all included studies, including baseline characteristics and outcome measures.

## Data Availability

The datasets used and analyzed during the current study are available from the corresponding author on reasonable request. The complete data extraction sheet is provided as Supporting File 1.
